# Using Virtual Reality to Assess Spatial Navigation Ability in Individuals With Mild Cognitive Impairment and Older Adults: Cross-Sectional Study

**DOI:** 10.2196/75952

**Published:** 2025-12-04

**Authors:** Ming-Chyi Pai, Yuh-Ting Lin, Chung-Yao Hsiao, Chia-Hung Lai, Chih-Jen Chen, Chaur-Jong Hu, Cheng-Yu Chen

**Affiliations:** 1 Division of Behavioral Neurology, Department of Neurology, National Cheng Kung University Hospital, College of Medicine, National Cheng Kung University, 138 Sheng-Li Road, North District, Tainan, 70428, Taiwan, 886 62353535 ext 5534, 886 62088036; 2 Alzheimer’s Disease Research Center, National Cheng Kung University Hospital, Tainan, Taiwan; 3 Institute of Gerontology, College of Medicine, National Cheng Kung University, Tainan, Taiwan; 4 Department of Medicine, National Cheng Kung University Hospital, Tainan, Taiwan; 5 Department of Neurology, Medical College, Taipei Medical University, Taipei, Taiwan; 6 Department of Neurology, Shuang-Ho Hospital, Taipei Medical University, New Taipei, Taiwan; 7 Department of Medical Imaging, Taipei Medical University Hospital, Taipei, Taiwan; 8 Department of Radiology, School of Medicine, Taipei Medical University, Taipei, Taiwan; 9 Translational Imaging Research Center, Taipei Medical University Hospital, Taipei, Taiwan; 10 Department of Radiology, National Defense Medical Center, Taipei, Taiwan; 11 Neuroscience Research Center, Taipei Medical University, Taipei, Taiwan

**Keywords:** virtual reality, mild cognitive impairment, spatial navigation, dementia, Alzheimer disease

## Abstract

**Background:**

Spatial navigation impairment is prevalent in people with Alzheimer disease (AD) and may appear in its initial clinical stage. Detecting this deficit in people at risk may not only help prevent them from getting lost or going missing but also provide a useful clinical aid to accurate diagnosis. Traditional assessments for spatial navigation impairment include questionnaires, paper-and-pencil and maze tests, or video games. While a real-world setting is more valid, direct, and accurate, it is plagued by unpredictable conditions such as weather, obstacles, or accidents. Owing to modern technology, virtual reality (VR) offers a new way to test spatial navigation impairment.

**Objective:**

The aims of this study were to test the feasibility of a VR setting to assess sense of location in people with mild cognitive impairment (MCI) and the power of VR to discriminate among groups with different clinical conditions.

**Methods:**

We used the Pai-Jan virtual reality (PJVR) device to test spatial navigation ability in those who were cognitively unimpaired (CU) and those who experienced subjective cognitive decline (SCD) and MCI. The PJVR device is the VR version (VIVE Pro Eye head-mounted display) of the Pai-Jan device, which has demonstrated its power to discriminate among CU, AD MCI, and mild AD dementia. With a map provided and using joysticks or handles, participants were asked to reach 5 points on a 660-m path. Linear deviation (LD; in meters) from each target point and vector deviation (in degrees) from the direction to the start point at each location were treated as the variables for comparison.

**Results:**

A total of 113 participants provided informed consent to initiate the study. Of these 113 participants, 93 (82.3%) completed the trials, including 22 (24%) who were CU, 39 (42%) with SCD, and 32 (34%) with MCI. In total, 17.7% (20/113) failed the trials due to cybersickness. The mean LD of the CU, SCD, and MCI groups was 38.2 (SD 39.5), 50.4 (SD 40.7), and 100.4 (SD 46.2) meters, respectively (*P*<.001). The MCI group showed greater vector deviation (mean 63.2, SD 42.4 degrees) than either the SCD (mean 39.4, SD 33.0 degrees) or CU (mean 38.6, SD 37.4 degrees; *P*=.02) group. The LD of the PJVR device was correlated with the total scores on the caregiver version of the Questionnaire on Everyday Navigational Ability (*P*<.001), indicating good ecological validity.

**Conclusions:**

The PJVR device is feasible for older adults and participants with MCI. It can detect spatial navigation deficits related to AD pathology, and the results show a close correlation with real-world navigation ability.

## Introduction

Spatial navigation impairment (SNI) is frequently encountered in people with Alzheimer disease (AD) and may appear in the early clinical stage of AD [1]. These patients usually compensate for their difficulties by using geographic or literal cues, taking fixed routes, or reducing their range of ambulation. As the disease advances, their ability to construct allocentric spatial representations and the translation between spatial reference frames becomes progressively impaired [2]. At this stage, they are no longer able to adopt alternatives when they are disoriented and may get lost [3,4]. Getting lost may result in going missing when efficient problem-solving strategies are absent or when timely help from pedestrians is unavailable. Although getting lost or going missing usually occurs in people with AD, it may also occur in those with neurodegenerative disorders due to non-AD pathology (MC Pai, unpublished data, April 2024). Under such conditions, the manifestations may be different from those of AD. People with Lewy body diseases, for example, may lose their way due to impaired egocentric spatial memory, executive dysfunction, or attentional deficit [5].

Impaired episodic memory is arguably a powerful indicator of hippocampal dysfunction and, hence, a valuable clinical feature for a diagnosis of AD. Notably, SNI or even getting lost may appear before episodic memory impairment in some patients with AD as one of the incipient symptoms [1]. In focal brain region models, the medial temporal lobe, especially the hippocampus, reflected the activity of allocentric navigation and episodic memory [6]. As the brain regions damaged by initial AD pathology overlap with the brain areas essential for human spatial navigation, SNI has been proposed as a marker for the diagnosis of AD [7-10]. Hence, the detection of SNI can not only prevent instances of getting lost or going missing but also identify early symptoms of clinical AD.

Currently, many tests have been developed to assess spatial navigation abilities. A real-world and large-scale setting is definitely more valid, direct, and accurate. However, unpredictable or unfavorable conditions may arise during testing, such as bad weather, physical discomfort, obstacles, or even accidents [11]. Nowadays, the various forms of virtual reality (VR) are mature and ready to assess spatial cognition in humans [12,13] (Table 1). For example, Tu et al [14] designed an indoor-environment VR test that was used to test spatial navigation abilities and episodic memory to distinguish AD from frontotemporal dementia, whereas Rekers and Finke [15] provided a novel design that reduced the confounding factors of fine motor skills to test the two abilities. However, many patients with AD lost their way in the outdoor environment, causing great life-threatening issues and social burden. Therefore, we hoped to design a large-scale virtual world to test SNI based on our previous model conducted in the real world.

**Table 1.** Examples of tests to assess spatial navigation ability.

Category	Examples
Function assessments	SBSOD^a^ [16] QuENA^b^ [17] WQ^c^ [18]
Pencil-and-paper or maze test	FMT^d^ [19]
Desktop VR^e^ test	CG^f^ Arena [20,21] SHQ^g^ [22] vYSA^h^ [23] The Navigation Test Suite [24,25]
Real-world settings	PJ^i^ test [26] DNT^j^ [27]
Immersive VR	VST^k^ [14] Triangle completion test [12] VIENNA^l^ [15] PJVR^m^ [13] VR Morris Water Maze [28] VENLab^n^ [29] Loop closure test [30] Y-maze task [31]
Others	TCTs^o^ [32] HGT^p^ [33]

a SBSOD: Santa Barbara Sense of Direction Scale.

b QuENA: Questionnaire on Everyday Navigational Ability.

c WQ: Wayfinding Questionnaire.

d FMT: floor maze test.

e VR: virtual reality.

f CG: computer generated.

g SHQ: Sea Hero Quest.

h vYSA: virtual Y-maze strategy assessment.

i PJ: Pai-Jan.

j DNT: detour navigation test.

k VST: virtual supermarket task.

l VIENNA: virtual environments navigation assessment.

m PJVR: virtual reality version of the Pai-Jan device.

n VENLab: Virtual Environment Navigation Lab.

o TCT: triangle completion task.

p HGT: hidden goal task.

As a result, the aims of this study were to test the feasibility of a VR setting to assess sense of location in people with mild cognitive impairment (MCI) and the power of VR to discriminate among groups with different clinical conditions. We hypothesized that the MCI group would have the poorest navigational performance compared to the cognitively unimpaired (CU) and subjective cognitive decline (SCD) groups, whereas the CU group would perform better than the SCD group.

## Methods

### Participants

We conducted a prospective study on individuals with cognitive impairment or concerns who regularly visited a special clinic in a university hospital. Participants included individuals with MCI and SCD and individuals who were CU. A diagnosis of MCI was made by a senior behavioral neurologist, MCP, based on patients’ clinical manifestations, the results of neuropsychological examinations, brain magnetic resonance imaging, and single-photon emission computed tomography [34]. Some participants received tests for AD biomarkers (Table 2). All participants with MCI met the Petersen criteria for MCI [35]: (1) memory concerns, preferably corroborated by an informant; (2) impaired memory function for their age and educational level; (3) preserved general cognitive function; (4) intact activities of daily living; and (5) absence of dementia. Similarly, a diagnosis of SCD was based on the chief concern of the patients and the scores on the Subjective Cognitive Decline Questionnaire (SCD-Q) [36].

**Table 2.** The results of plasma pTau217 levels and amyloid positron emission tomography (PET).

	CU^a^ (n=22), n (%)	SCD^b^ (n=39), n (%)	MCI^c^ (n=32), n (%)	Total (N=93), n (%)
Plasma pTau217				
Low level	10 (45)	12 (31)	7 (22)	29 (31)
Medium level	1 (5)	3 (8)	3 (9)	7 (8)
High level	3 (14)	4 (10)	15 (47)	22 (24)
Amyloid PET				
Negative	1 (5)	17 (44)	2 (6)	20 (22)
Positive	0 (0)	1 (3)	2 (6)	3 (3)
Unknown	7 (32)	2 (5)	3 (9)	12 (13)
ADNC^d^	3 (14)	5 (13)	17 (53)	25 (27)

a CU: cognitively unimpaired.

b SCD: subjective cognitive decline.

c MCI: mild cognitive impairment.

d ADNC: Alzheimer disease neuropathologic changes, including high plasma pTau217 level or positive amyloid PET.

Because of the novelty of using this device to quantify spatial disorientation and the absence of established normative benchmarks for key outcome measures such as linear deviations (LDs) and vector deviations (VDs), a priori sample size calculation was based on effect sizes reported in related literature [13]. Assuming a large effect size (Cohen *f*=0.40), a significance level of .05, and a statistical power of 80%, we estimated that a total sample size of 84 participants would be sufficient to detect statistically significant group differences using one-way ANOVA. To account for potential exclusions due to VR-related adverse effects or incomplete data, we planned for an initial recruitment target of 90 to 100 participants, anticipating an attrition rate of up to 20%.

### Basic Measurements

Basic measurements included the Cognitive Abilities Screening Instrument [37], Clinical Dementia Rating Scale [38], Geriatric Depression Scale [39], SCD-Q [36], and Questionnaire on Everyday Navigational Ability (QuENA) [17]. The QuENA consists of patient (QuENA-P) and caregiver (QuENA-CG) versions. The definition of caregivers in this study was the spouses, children, or key care partners of the participants. We used perceived ease of use [40], the Computer Anxiety Scale [41], and perceived enjoyment [42,43] to assess domains from the technology acceptance model (TAM). Moreover, we inquired and recorded any symptoms of cybersickness, including nausea, oculomotor discomfort, and disorientation. Most of the questionnaires were administered before the VR test, whereas several questions regarding cybersickness and technological acceptance were administered after the test to find out the feasibility and practicality of this device.

### Assessment of Spatial Navigation Abilities

**Figure 1.**
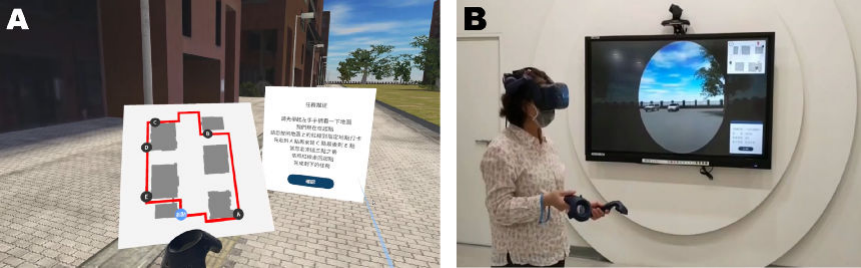
The Pai-Jan virtual reality test. The figure shows (A) a map in the virtual space and the blue light beam for functional manipulation and (B) the testing environment from the examiner's views. The map clearly demonstrated 5 spots, A, C, E, D, and B, and the light blue spot represented the starting point. Participants could read written instructions on a billboard, and at the same time, the recording repeated the words through earphones on the head-mounted display.

As indicated in our previous research [13], we remodeled the Tzu-Chiang campus of National Cheng Kung University to adapt it to our VR devices due to its ecological validity and for further comparisons. The Pai-Jan virtual reality (PJVR) device set included a VIVE Pro Eye head-mounted display, joysticks, and sensors, which were contract-manufactured by HTC International Corporation, whereas the software was from SteamVR (Valve) and the Unity game engine (Unity Technologies). In the pretest phase, the participants were instructed to approach the designated punch-in points A, C, and E in the first round and D and B in the second round by manipulating joysticks, which allowed them to move forward and backward, punch in, and select the answers to the questions. The selection of punch-in points was based on visual blockage from the buildings in this model (Figure 1). The other scenario details, such as the exterior of the buildings, the texture of the asphalt road, and the addition of trees, cars, and other materials, strived to be the same as those of the real world. Two parameters, LD and VD, were tested in this study. LD was defined as a relative value (in meters) based on the coordinates established in the system determining the degree of difference between the location where the participant punched in and the correct punching point. VD was defined as the angular difference (in degrees) between 2 vectors originating from the starting point: one connecting the starting point to the target checkpoint (the correct location) and the other representing the participant’s pointing direction toward the presumed start point. LD was tested using the computer system, which calculated the relative distance between the standing point of the participants in the VR world and the correct punch-in point. We also asked the participants to point at the starting point (Chi-Mei building, the building with white exterior), and the computer determined the angular differences through the in-built system. The VR tests concluded after 2 rounds, and the remaining questionnaires mentioned previously were then administered. All the examinations were conducted in a classroom with great care for security and comfort to reduce other factors that may affect the results of the tests.

### Data Analysis and Statistics

All data were properly stored in an ethical manner. Continuous variables are presented as means and SDs, whereas categorical variables are summarized as counts and percentages. To compare demographic characteristics across the 3 groups, we first assessed the normality of the continuous variables. On the basis of the results, one-way ANOVA was applied to variables with a normal distribution, whereas the Kruskal-Wallis test was used for those that did not meet the normality assumption. For categorical variables, the chi-square test was used to examine group differences when expected cell counts were adequate. When the overall group comparison was statistically significant, post hoc pairwise comparisons were conducted using least significant difference to identify specific between-group differences. A correlation was analyzed between the total QuENA score and LD and VD. The main purpose of this study was not to concentrate on over- or underestimation judgments of distance or vector, so the VDs in the statistical analysis were processed in absolute values.

All statistical analyses were conducted using SPSS (version 17.0; IBM Corp), with a 2-sided significance level of α=.05.

### Ethical Considerations

This study was approved by the ethical review board of National Cheng Kung University Hospital located in Tainan, Taiwan (B-BR-110-046). All participants gave their written informed consent to take part. The data were deidentified during analytic processes and were stored in an ethical and secure manner to prevent data leakage.

## Results

A total of 128 participants provided informed consent. However, of these 128 participants, 15 (11.7%) were not tested using the PJVR device because 10 (67%) changed their minds and 5 (33%) had medical issues. Of the 113 participants who initiated the trials, 20 (17.7%) developed cybersickness and were unable to complete the PJVR test, including 4 (20%) who were CU, 7 (35%) with SCD, and 9 (45%) with MCI. Eventually, 93 participants finished the procedure, including 22 (24%) who were CU, 39 (42%) with SCD, and 32 (34%) with MCI. The demographic data are shown in Table 3.

**Table 3.** Demographic data and basic assessments. Least significant difference tests were conducted as the post hoc test.

	CU^a^ (n=22)	SCD^b^ (n=39)	MCI^c^ (n=32)	*F* test (*df*)	*P* value	Post hoc test	95% CI of the mean
Sex (male), n (%)	12 (55)	22 (56)	15 (47)	—^d^	.71	ns^e^	—
Age (y), mean (SD)	67.7 (5.7)	68.5 (4.2)	70.0 (3.7)	1.818 (2, 90)	.17	ns	67.9-69.8
Education (y), mean (SD)	13.3 (1.7)	13.1 (2.5)	12.0 (3.1)	2.355 (2, 90)	.10	ns	12.2-13.3
CDR^f^ (n=15/15/32; range 0-3), mean (SD)	0.1 (0.2)	0.3 (0.2)	0.5 (0.0)	23.579 (2, 90)	<.001	MCI>SCD>CU	0.3-0.4
GDS^g^ (range 0-15), mean (SD)	0.5 (0.7)	0.5 (1.0)	0.7 (1.6)	0.204 (2, 90)	.82	ns	0.3-0.8
SCD-Q-P^h^ (range 0-24), mean (SD)	5.2 (3.2)	9.0 (5.4)	8.2 (5.0)	4.622 (2, 90)	.01	MCI=SCD>CU	6.8-8.9
SCD-Q-CG^i^ (range 0-24), mean (SD)	4.8 (4.8)	9.7 (5.0)	14.3 (2.6)	26.742 (2, 90)	<.001	MCI>SCD>CU	9.2-11.9
MMSE^j^^,^^k^ (range 0-30), mean (SD)	27.5 (1.9)	28.1 (1.6)	22.7 (2.9)	58.953 (2, 90)	<.001	CU=SCD>MCI	25.4-26.8
CASI^l^ total score (range 0-100), mean (SD)	91.8 (4.3)	92.5 (3.8)	77.7 (9.5)	53.769 (2, 90)	<.001	CU=SCD>MCI	85.3-89.2
Remote memory	10.0 (0.0)	10.0 (0.3)	9.6 (0.9)	3.584 (2, 90)	.03	CU=SCD>MCI	9.7-9.9
Recent memory	10.4 (1.3)	11.2 (1.0)	6.3 (3.0)	59.903 (2, 90)	<.001	CU=SCD>MCI	8.7-10.0
Attention	7.7 (0.6)	7.7 (0.6)	7.2 (0.9)	4.999 (2, 90)	.009	CU=SCD>MCI	7.4-7.7
Mental manipulation	9.2 (1.3)	9.2 (1.2)	8.4 (2.2)	2.447 (2, 90)	.09	SCD>MCI	8.6-9.3
Orientation	17.8 (0.5)	17.9 (0.3)	13.8 (3.6)	38.021 (2, 90)	<.001	CU=SCD>MCI	15.9-17.1
Abstract thinking	8.6 (1.5)	8.6 (1.5)	7.7 (1.3)	4.911 (2, 90)	.009	CU=SCD>MCI	8.0-8.6
Language	9.7 (0.5)	9.9 (0.4)	9.6 (0.8)	2.675 (2, 90)	.07	SCD>MCI	9.6-9.8
Drawing	9.9 (0.4)	9.6 (0.8)	8.8 (1.5)	7.374 (2, 90)	.001	CU=SCD>MCI	9.2-9.6
Verbal fluency	8.5 (2.0)	8.5 (1.7)	6.3 (2.3)	12.073 (2, 90)	<.001	CU=SCD>MCI	7.3-8.2
QuENA^m^ (range 0-30), mean (SD)							
QuENA-P^n^ total	1.6 (1.6)	3.6 (3.1)	4.0 (3.4)	4.728 (2, 90)	.01	MCI=SCD>CU	2.7-3.9
LSA^o^	0.1 (0.4)	1.1 (1.5)	1.1 (1.4)	4.432 (2, 90)	.02	MCI=SCD>CU	0.6-1.1
ED^p^	0.2 (0.4)	0.5 (0.9)	0.9 (1.1)	3.54 (2, 90)	.03	MCI>CU	0.4-0.8
INA^q^	1.0 (1.0)	1.6 (1.1)	1.2 (1.3)	1.974 (2, 90)	.15	ns	1.1-1.6
HD^r^	0.2 (0.4)	0.4 (0.8)	0.9 (1.3)	3.668 (2, 90)	.03	MCI>CU=SCD	0.3-0.8
QuENA-CG^s^ total	2.3 (2.1)	3.2 (2.8)	5.7 (4.1)	6.492 (2, 90)	.003	MCI>CU=SCD	3.3-5.0
LSA	0.1 (0.4)	0.7 (1.0)	1.4 (1.6)	6.271 (2, 90)	.003	MCI>CU=SCD	0.6-1.2
ED	0.5 (0.9)	0.7 (1.0)	1.4 (1.3)	3.944 (2, 90)	.02	MCI>CU=SCD	0.7-1.2
INA	1.5 (1.0)	1.2 (1.0)	1.5 (1.6)	0.354 (2, 90)	.70	ns	1.1-1.7
HD	0.3 (0.6)	0.7 (1.2)	1.5 (1.5)	5.247 (2, 90)	.008	MCI>CU=SCD	0.6-1.3

a CU: cognitively unimpaired.

b SCD: subjective cognitive decline.

c MCI: mild cognitive impairment.

d Not applicable.

e ns: not significant.

f CDR: Clinical Dementia Rating Scale.

g GDS: Geriatric Depression Scale.

h SCD-Q-P: patient version of the Subjective Cognitive Decline Questionnaire.

i SCD-Q-CG: caregiver version of the Subjective Cognitive Decline Questionnaire.

j MMSE: Mini-Mental State Examination.

k Extracted and modified from the Cognitive Abilities Screening Instrument.

l CASI: Cognitive Abilities Screening Instrument.

m QuENA: Questionnaire on Everyday Navigational Ability.

n QuENA-P: patient version of the QuENA.

o LSA: landmark and scene agnosia.

p ED: egocentric agnosia.

q INA: inattention.

r HD: heading disorientation.

s QuENA-CG: caregiver version of the QuENA.

Table 4 shows the results for the TAM domains and cybersickness. Regarding the TAM, the CU group expressed more enjoyment than the SCD group, whereas the MCI group manifested less perceived ease of use than the other 2 groups, which warrants further investigation. Regarding cybersickness, no difference was detected in any symptoms among the 3 groups. This is an important finding for future application.

**Table 4.** Technology acceptance and cybersickness results. Least significant difference tests were conducted for the post hoc test.

Item	CU^a^ (n=22), mean (SD)	SCD^b^ (n=39), mean (SD)	MCI^c^ (n=32), mean (SD)	*F* test (*df*)	*P* value	Post hoc test	95% CI of the mean
PEOU^d^ 1	6.0 (1.1)	6.0 (1.1)	5.0 (1.9)	5.723 (2, 90)	.005	CU=SCD>MCI	5.4-6.0
PEOU 2	5.1 (2.0)	4.9 (2.3)	5.0 (1.7)	0.117 (2, 90)	.89	ns^e^	4.6-5.4
PEOU 3	6.0 (0.9)	5.3 (1.3)	4.9 (1.7)	4.468 (2, 90)	.01	CU>MCI	5.0-5.6
PEOU 4	5.9 (1.0)	5.4 (1.5)	5.0 (1.6)	2.228 (2, 90)	.11	ns	5.1-5.7
CANX^f^ 1	7.0 (0.2)	6.3 (1.8)	6.1 (2.1)	1.916 (2, 90)	.15	ns	6.0-6.7
CANX 2	6.1 (1.8)	5.2 (2.2)	5.2 (2.2)	1.712 (2, 90)	.19	ns	5.0-5.8
CANX 3	5.5 (2.1)	5.2 (2.2)	5.8 (1.9)	0.891 (2, 90)	.41	ns	5.1-5.9
CANX 4	6.7 (1.3)	6.1 (1.8)	6.1 (1.8)	0.907 (2, 90)	.41	ns	5.9-6.6
ENJ^g^ 1	5.3 (1.5)	4.4 (1.7)	4.8 (1.6)	2.164 (2, 90)	.12	ns	4.4-5.1
ENJ 2	5.5 (1.1)	4.6 (1.5)	5.1 (1.4)	3.553 (2, 90)	.03	CU>SCD	4.7-5.3
ENJ 3	5.6 (1.2)	4.7 (1.4)	5.0 (1.6)	3.132 (2, 90)	.048	CU>SCD	4.7-5.3
Nausea	17.8 (26.5)	21.3 (32.2)	7.8 (12.5)	2.540 (2, 90)	.08	ns	10.4-21.2
Oculomotor discomfort	8.3 (11.7)	18.1 (27.0)	9.7 (20.3)	1.914 (2, 90)	.15	ns	8.3-17.4
Disorientation	18.3 (29.3)	33.6 (59.2)	16.5 (30.1)	1.518 (2, 90)	.23	ns	14.9-33.3
Total score	16.0 (23.3)	26.3 (40.7)	12.3 (22.0)	1.868 (2, 90)	.16	ns	12.5-25.6

a CU: cognitively unimpaired.

b SCD: subjective cognitive decline.

c MCI: mild cognitive impairment.

d PEOU: perceived ease of use.

e ns: not significant.

f CANX: Computer Anxiety Scale.

g ENJ: perceived enjoyment.

### Findings of the Core Test

The MCI group took more time to complete the test than the SCD or CU groups in the counterclockwise A-C-E sequence, but no difference was detected in the clockwise B-D sequence.

The mean LD of the CU, SCD, and MCI groups was 38.2 (SD 39.5), 50.4 (SD 40.7), and 100.4 (SD 46.2) meters, respectively (*P*<.001; Table 5). LD showed a large group effect (*F*_2, 90_=17.87; *P*<.001; ω^2^=0.27; partial η^2^=0.28; *f*=0.63). In the same way, the MCI group yielded a greater VD than either the SCD or CU group (*P*=.02). VD showed a small to medium group effect (*F*_2, 90_=4.33; *P*=.02; partial η^2^=0.088; ω^2^=0.067; Cohen *f*=0.31).

**Table 5.** Time to complete the test, linear deviation, and vector deviation. A, B, C, D, and E indicate the 5 points in the setting. Least significant difference tests were conducted for the post hoc test.

	CU^a^ (n=22), mean (SD)	SCD^b^ (n=39), mean (SD)	MCI^c^ (n=32), mean (SD)	*F* test (*df*)	*P* value	Post hoc test	95% CI of the mean
Time to complete (min)							
A-C-E	23.4 (8.8)	25.5 (7.4)	32.7 (11.7)	7.855 (2, 90)	.001	MCI>CU=SCD	25.4-29.5
B-D	19.3 (4.6)	19.8 (6.1)	22.5 (10.2)	1.524 (2, 90)	.22	ns^d^	19.1-22.2
Linear deviation (m)							
A	31.9 (61.2)	25.4 (45.7)	92.3 (80.1)	11.088 (2, 90)	<.001	MCI>CU=SCD	35.6-64.3
B	29.5 (45.4)	54.7 (66.9)	116.4 (64.8)	14.920 (2, 90)	<.001	MCI>CU=SCD	55.5-84.8
C	66.5 (93.1)	90.2 (94.7)	131.7 (101.6)	3.229 (2, 90)	.04	MCI>CU	78.5-119.3
D	22.2 (24.3)	30.4 (35.5)	63.5 (50.9)	9.050 (2, 90)	<.001	MCI>CU=SCD	31.0-48.7
E	41.3 (55.5)	49.3 (54.4)	98.3 (75.3)	7.314 (2, 90)	.001	MCI>CU=SCD	50.5-78.0
Mean	38.2 (39.5)	50.4 (40.7)	100.4 (46.2)	17.866 (2, 90)	<.001	MCI>CU=SCD	54.5-74.9
Vector deviation (degrees)							
A	26.3 (58.0)	20.1 (42.3)	67.7 (93.8)	4.799 (2, 90)	.01	MCI>CU=SCD	23.5-52.4
B	42.4 (56.5)	42.2 (53.4)	66.9 (83.6)	1.447 (2, 90)	.24	ns	37.1-64.6
C	46.8 (92.1)	37.7 (51.4)	47.8 (50.7)	0.271 (2, 90)	.76	ns	30.4-56.2
D	40.3 (66.9)	34.8 (64.0)	49.9 (54.7)	0.533 (2, 90)	.59	ns	28.6-53.9
E	37.0 (48.7)	61.1 (66.3)	83.4 (81.6)	3.015 (2, 90)	.05	ns	48.6-77.5
Mean	38.6 (37.4)	39.4 (33.0)	63.2 (42.4)	4.333 (2, 90)	.02	MCI>CU=SCD	39.4-55.3

a CU: cognitively unimpaired.

b SCD: subjective cognitive decline.

c MCI: mild cognitive impairment.

d ns: not significant.

The LD of the PJVR device was correlated with the total scores on the QuENA-CG (*P*<.001), indicating good ecological validity. The correlation with the scores on the QuENA-P was not significant (*P*=.07). As expected, the MCI group yielded a larger LD and VD than the other groups in judging their current location and path integration, the functions of which are highly related to the entorhinal cortex [44].

## Discussion

### Feasibility

The PJVR device is theory-driven and carefully designed to detect deficits in spatial cognition in people with cerebral AD pathology [13,26]. The fact that 82.3% (93/113) of the participants completed the procedures with satisfactory technology acceptance supports its feasibility for use in older adults and individuals with MCI. Our recent research showed that people at the stage of all-cause MCI were at risk of getting lost or going missing. Hence, a test to detect SNI in this group is important. This study is part of the limited research that has used an immersive VR setting to assess spatial cognition in MCI in Asian countries, and the results may encourage more researchers to develop similar devices in this field.

### Principal Findings

The CU and SCD groups shared similar features, mostly in demographics, cognitive assessment, and functions of sense of location and path integration. The SCD group was similar to the MCI group in SCD-Q and QuENA-P scores as these 2 questionnaires were self-reported by patients and the SCD group may have downplayed their spatial ability and exaggerated their cognitive impairment out of anxiety.

**Figure 2.**
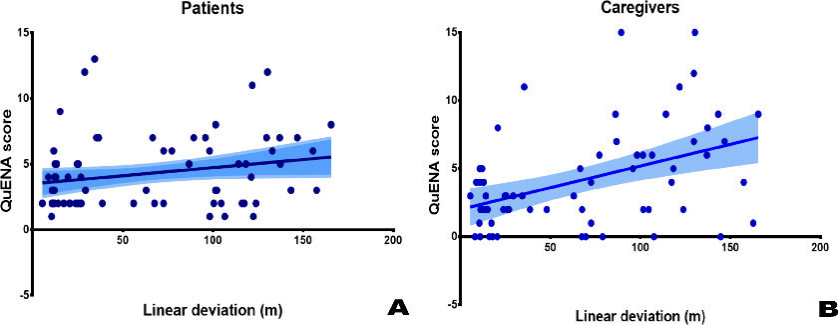
The correlation between the Questionnaire on Everyday Navigational Ability (QuENA) total score and linear deviation: (A) total score on the patient version of the QuENA (0.01232 × linear deviation [meters] + 3.493; *R*^2^=0.0483; *P*=.07) and (B) total score on the caregiver version of the QuENA (0.03180 × linear deviation [meters] + 2.020; *R*^2^=0.1968; *P*<.001).

As expected, the MCI group spent more time completing the test as their spatial navigation ability was worse than that of the other 2 groups (Table 5). The correlation was high between the LD and the QuENA-CG score but not between the LD and the QuENA-P score (Figure 2). The QuENA-CG assesses explicit navigation behavior observed by others and shows good predictive accuracy for risk of getting lost [17,45]. Hence, it reflects the SNI of these patients and is useful for identifying people at risk of getting lost or going missing. However, the MCI group may have overestimated their navigational ability, and the greater the overestimation, the greater the likelihood of getting lost [17,45]. This may explain the poor correlation between the LD and the QuENA-P score. This was also true for the correlation between VD and the total scores on the QuENA-P and QuENA-CG.

In terms of the comparison between the CU and SCD groups, both showed similar navigational abilities and mental functions, which were supported by the LD and VD results and the Cognitive Abilities Screening Instrument scores in our study. However, in the QuENA-P, the SCD group showed similar patterns of getting lost to those of patients with MCI in several aspects.

The absence of a difference in LD or VD between the CU and SCD groups did not support our hypothesis. One of the reasons is that SNI does not appear before the clinical stage of MCI. However, this was a cross-sectional study rather than a longitudinal one, so it was not possible to recognize the gradual deterioration of navigational abilities in patients with MCI who had been through the CU and SCD periods. Another possibility is that there was a difference in SNI between these 2 groups, but the current assessment was not sensitive enough to detect it. On the other hand, the SCD group showed mismatched results in comparison with patients with MCI in subjective getting lost experience and the standardized PJVR testing environment, which may be due to distinct severity of spatial disorientation.

### Clinical Implications

Daily navigation in a familiar environment usually takes only a few minutes to half an hour. When traveling from one place to another, events that occur along the way may distract the navigator. Keeping the destination or sequential stops along the journey in mind is a challenge to those with poor spatial orientation and episodic memory. However, traditional neuropsychological assessments are not good at predicting the risk of getting lost or going missing for individuals at the stage of MCI or very mild AD dementia [3].

The QuENA examines navigation deficits in daily life explicitly and is superior to many similar questionnaires [17,46,47]. In this study, the correlations between the QuENA-CG total score and both LD and VD on the PJVR device were significant (Figures2  3; for LD, *P*<.001; for VD, *P*=.03). On the basis of this, for those with poor performance on the PJVR device, the risk of getting lost or going missing is high when timely help from others is unavailable and their problem-solving ability is also impaired. The definition of poor performance on the PJVR device using LD or VD cutoff values needs further study to verify its external validity.

**Figure 3.**
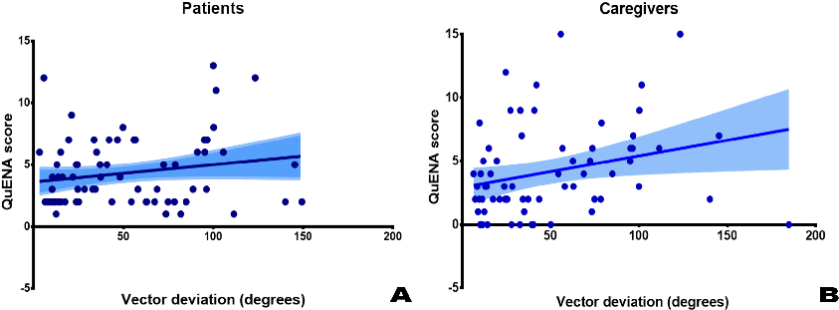
The correlation between the Questionnaire on Everyday Navigational Ability (QuENA) total score and vector deviation: (A) total score on the patient version of the QuENA (0.01391 × vector deviation [degrees] + 3.607; *R*^2^=0.03648; *P*=.11) and (B) total score on the caregiver version of the QuENA (0.02469 × vector deviation [degrees] + 2.943; *R*^2^=0.07384; *P*=.03).

The QuENA-CG assesses the risk of getting lost or going missing [45], and the PJVR device simulates real-world navigation scenarios. Thus, a poor performance on the latter indicates a higher risk of getting lost or going missing. However, different people may adopt different methods to overcome their disorientation and prevent themselves from going missing. Most older adults, for example, use an egocentric instead of an allocentric strategy to compensate for the impaired hippocampal function [47]. In this regard, the QuENA-CG score shows the risk of getting lost or going missing, and the PJVR device reveals evidence of SNI.

From a clinical viewpoint, patients are often aware of their deficits in spatial navigation but are unwilling to report the problem to others because of pride. Another possibility is that they cannot retain these disorientation or getting lost experiences in memory long enough to inform others. Gradually, difficulties in wayfinding increase until getting lost or going missing eventually occurs. Studies have revealed that baseline spatial navigation ability can predict clinical progression in patients in their midlife or with cognitive impairment [20,48-50]. Thus, we recommend a test or a questionnaire, such as the PJVR device or the QuENA, to assess spatial navigation ability in people with cognitive impairment, especially for those with suspected cerebral amyloid pathology. Moreover, many investigators have recommended SNI as a marker for diagnosing early AD [7-10,26]. Modification of the PJVR device to a simpler version is needed because the era of unsupervised digital cognitive assessment is imminent.

### Comparison With Other Similar VR Settings

In comparison with other immersive VR settings used to evaluate spatial navigation ability, the PJVR device has several advantages. First, it was remodeled from the real-world Pai-Jan device, and head-to-head comparison is possible. During the PJVR test, participants wear VR headsets and orient themselves by turning their heads, and navigation is typically performed using a monitor and joystick. However, due to space limitations, participants used handheld controllers to move rather than walking physically. As a result, proprioceptive input such as joint position and actual horizontal displacement was not engaged, making the PJVR device relatively less immersive in this respect compared to the original real-world Pai-Jan device or other tasks conducted in smaller physical spaces where participants can walk, such as the triangle completion test [12] or Morris Water Maze [28] or setups such as the Virtual Environment Navigation Lab [29]. Second, as a VR spatial navigation test, the PJVR system features a testing environment modeled after a real university campus, enhancing its ecological validity compared to other VR-based navigation tasks such as the triangle completion test [12], Morris Water Maze [28], loop closure test [30], or Y-maze task [31]. These settings are computer-generated or indoors or involve nature or wild scenarios rather than human environments. As mentioned previously, an outdoor scenario is more akin to real-world environments where people with cognitive impairment would get lost or go missing.

Third, the PJVR device is a theory-driven design based on neuroscience evidence and safer than real-world outdoor tasks. The PJVR device assesses not only distance errors during navigation but also sense of direction (eg, the ability to return to the starting point) and navigational efficiency (measured via completion time). As participants must pass through multiple checkpoints in sequence, navigation errors can accumulate progressively. In other words, the PJVR device captures sequential errors that occur during long-route spatial navigation, which is an aspect that is rarely examined in other VR-based navigation tasks. Given the spatial complexity of the PJVR environment, executive functions may also be required for task completion. To reduce the cognitive demands related to direction selection and route planning, participants follow a predefined path. Notably, the design ensures that checkpoints are obstructed by buildings, preventing direct visual access. This reduces reliance on landmark or beacon cues and visuospatial memory, thereby emphasizing the use of allocentric spatial navigation strategies. Finally, similar to other VR settings, the PJVR device is a standardized, well-designed program, and the researchers could record parameters of interest precisely for analysis. More detailed comparisons can be found in a book to be published [51].

In summary, the PJVR device primarily evaluates allocentric spatial navigation ability, achieves a notable degree of ecological validity, and measures multiple dimensions of spatial navigation performance. Its unique design allows for the detection of more detailed navigational behaviors. With appropriate application, the PJVR device holds potential for advancing new discoveries or the development of novel hypotheses in spatial cognition and dementia research.

### Limitations

In this study, participants with MCI were carefully selected based on clinical manifestation, neuropsychological tests, and neuroimages. Regarding the concept of early and late MCI, participants with late MCI might show worse performance in sense of location as assessed using the PJVR device. However, the number of participants in this study was insufficient to divide them into early and late MCI groups. Future studies with more participants and using amyloid positron emission tomography scans or fluid biomarkers to verify cerebral amyloid pathology are needed to answer this question. In addition, due to the innovation of our study, only a few studies could serve as the foundation, which might lead to insufficient explanatory power of the results. Nevertheless, this research provides a protocol for future studies to refer to, and the results presented in this paper could be verified by other researchers.

In conclusion, the PJVR device is feasible for assessing spatial navigation ability in people with MCI and can distinguish them from those who are CU or with SCD. Poor performers in the PJVR test have a higher risk of getting lost or going missing, and their care partners must take action to prevent unfavorable events.

## Abbreviations

AD: Alzheimer disease
CU: cognitively unimpaired
LD: linear deviation
MCI: mild cognitive impairment
PJVR: Pai-Jan virtual reality
QuENA: Questionnaire on Everyday Navigational Ability
QuENA-CG: caregiver version of the Questionnaire on Everyday Navigational Ability
QuENA-P: patient version of the Questionnaire on Everyday Navigational Ability
SCD: subjective cognitive decline
SCD-Q: Subjective Cognitive Decline Questionnaire
SNI: spatial navigation impairment
TAM: technology acceptance model
VD: vector deviation
VR: virtual reality
